# Editorial: Advances in the pharmacotherapy of chronic pelvic pain conditions

**DOI:** 10.3389/fphar.2025.1546059

**Published:** 2025-01-29

**Authors:** Elena Lucarini, Javier Aguilera-Lizarraga, Miguel Ángel Tejada

**Affiliations:** ^1^ Department of Neuroscience, Psychology, Drug Research and Child Health, NEUROFARBA, Section of Pharmacology and Toxicology, University of Florence, Florence, Italy; ^2^ Department of Pharmacology, University of Cambridge, Cambridge, United Kingdom; ^3^ Department of Pharmacology, Faculty of Medicine, University of Granada, Granada, Spain; ^4^ Department of Pharmacology, Biomedical Research Center, Institute of Neuroscience, University of Granada, Granada, Spain; ^5^ Department of Pharmacology, Instituto de Investigación Biosanitaria de Granada (ibs.GRANADA), Granada, Spain

**Keywords:** chronic pelvic pain, inflammatory bowel disease, irritable bowel syndrome, vulvodynia, endometriosis, prostatitis

Chronic pelvic pain (CPP) is a multifactorial condition, which affects around 26% of the global population, predominantly women (Lamvu et al., 2021), and strongly impacts patients’ quality of life (Lamvu et al., 2021; Daniels and Khan, 2010). The pathophysiology of CPP is complex, involving various underlying causes such as gastrointestinal, gynecological or urological diseases (Horne and Missmer, 2022; Paspulati, 2023). Despite its high prevalence, current treatments have limited effectiveness, highlighting the urgent need for individualized therapies. To this end, a better understanding of the pathophysiological mechanisms underlying CPP is required.

Recent years have seen notable advancements in the comprehension and management of this condition, driven by interdisciplinary research and clinical innovation. One significant breakthrough has been the adoption of a multimodal approach, integrating medical, psychological, and physical therapies tailored to individual needs. Advances in imaging and diagnostic tools, such as high-resolution pelvic MRIs and laparoscopic techniques, have also illuminated potential underlying mechanisms. In this Research Topic, we aimed to gather new and suitable pharmacological approaches for the management of pelvic pain, along with functional and psychiatric comorbidities, exploring the pathways involved in the physiology of viscera-brain axis and identifying novel therapeutic targets. There were included 5 *original research articles* and one *clinical trial*. Starting with the formers, Fusco et al. evaluated the efficacy of combining *Acmella oleracea* and *Boswellia* serrata extracts (AO + BS) for the treatment of vulvodynia-like symptoms. To this end, they used a mouse model of disease by the repeated injection the pro-inflammatory Complete Freund’s adjuvant (CFA) into the distal vaginal. In addition to provide acute relief from mechanical pain, the treatment with AO+ BS significantly reduced pain persistence, spinal microgliosis and neuronal overexcitation associated with vulvodynia. Thus, AO and BS emerge as valid candidates for the management of chronic pain conditions like vulvodynia. Further studies will be needed to identify the mechanisms of action of these compounds and to define the best protocol to translate into clinical practice.

Lucarini et al. investigated the beneficial effects of a product containing gamma-aminobutyric acid and *Melissa officinalis* (GABA-Mo 5:1) on post-inflammatory irritable bowel syndrome induced by dinitrobenzene sulfonic acid (DNBS) injection in rats. Two different protocols of treatment were studied to assess either the preventive or the curative potential of GABA-Mo. In the preventive approach, GABA-Mo led to a significant reduction of spinal microglia reactivity, normalizing several parameters altered by inflammatory damage at both the colon (malondialdehyde and IL-1β increase, claudin-1 decrease) and plasma (lipopolysaccharide binding protein) levels. However, the curative approach was partially effective on pain. Here, GABA-Mo effectively counteracted pain persistence in DNBS-treated animals, reducing astrogliosis in the spinal cord and promoting colon healing from damage. These results suggest that supplementation with GABA-Mo might represent a suitable approach to manage chronic or recurrent abdominal pain resulting from inflammatory bowel damage. Although the authors suggested that strengthening integrity of the intestinal barrier might underlie the therapeutic effects of GABA-Mo, further mechanistic studies are required to confirm this.

Coxon et al. investigated clinical predictors of treatment response to gabapentin in women with unexplained CPP. Gabapentin, commonly used for neuropathic pain, has shown variable effectiveness in CPP cases. This study evaluated factors that may predict treatment success, including pain characteristics, psychological profiles, and comorbidities. Findings suggested that women with predominant neuropathic pain features, such as burning or shooting sensations, and those with lower levels of anxiety and depression, are more likely to benefit from gabapentin. Conversely, patients with centralized pain syndromes or significant psychological distress may experience limited improvement. The study emphasizes the importance of individualized therapies and suggests that identifying these predictors might improve the selection process for effective CPP management using gabapentin.

Wang et al. examined how evodiamine, a natural alkaloid with anticancer and anti-inflammatory properties, can inhibit the development of endometriosis induced by early exposure of mice to Epstein-Barr virus (EBV). The study identified estrogen receptor beta (ERβ) as a key factor in the progression of this condition following EBV infection. Evodiamine suppressed ERβ expression, reducing cell proliferation and inflammatory stress associated with endometriosis. Thus, researchers of this study demonstrated that this compound holds significant therapeutic potential for managing EBV-related endometriosis. The findings highlight a novel connection between EBV, ERβ, and endometriosis, offering insights for more targeted treatment approaches.

Liu et al. explored the anti-inflammatory and anti-nociceptive roles of Ningmitai capsules, containing a combination of herbs used in Chinese medicine, in pain associated with prostatitis. Using a non-obese diabetic mouse model (NOD/ShiLtJ) of prostate inflammation, upon autoimmunization with male accessory gland extracts in CFA, the authors showed that Ningmitai treatment reduced prostate inflammation and nociceptive responses to von Frey filaments (applied to the pelvis, abdomen, and paws). Moreover, systemic and prostate levels of CD11b^+^Ly6C^high^ myeloid cells—deemed pro-inflammatory—were reduced in Ningmitai-treated animals. Their findings also suggested a potential effect of Ningmitai capsules on the inflammatory CCL2 pathway, but additional studies are required to further understand the underlying mechanisms. Moreover, whether this treatment is effective in alleviating pelvic pain, rather than somatic pain in the pelvic area, requires further confirmation.

Finally, Shah et al. performed a single-centre, randomized, double-blind, positive-controlled Phase II clinical trial to test the additive effects of Fuke Qianjin (another combination of herbs used in Chinese medicine) to metronidazole and doxycycline hyclate treatment in pelvic inflammatory disease. The authors showed that Fuke Qianjin supplementation had an acceptable safety profile and tolerance. However, this treatment proved to have similar efficacy when compared to the control group (i.e., patients receiving only antibiotics), and hence concluded that Fuke Qianjin supplementation was non-inferior to the control. Whether a longer treatment duration or different doses yield greater benefits remains to be elucidated.

In conclusion, this Research Topic included 5 *original research articles* and one *clinical trial*. All of these fit the scope of this Research Topic titled “Advances in the Pharmacotherapy of Chronic Pelvic Pain Conditions,” which aimed to explore advancements in pharmacotherapy, focusing on emerging treatments, clinical findings, and their implications for improving patient outcomes in the management of chronic pelvic pain. These developments offer hope for more effective, personalized approaches to treating this complex and often misunderstood condition.

## References

[B1] Daniels J. P. Khan K. S. (2010). Chronic pelvic pain in women. BMJ 341, c4834. 10.1136/bmj.c4834 20923840

[B2] Horne A. W. Missmer S. A. (2022). Pathophysiology, diagnosis, and management of endometriosis. BMJ 379, e070750. 10.1136/bmj-2022-070750 36375827

[B3] Lamvu G. Carrillo J. Ouyang C. Rapkin A. (2021). Chronic pelvic pain in women: a review. JAMA - J. Am. Med. Assoc. 325, 2381–2391. 10.1001/jama.2021.2631 34128995

[B4] Paspulati R. M. (2023). Chronic pelvic pain: role of imaging in the diagnosis and management. Seminars Ultrasound, CT MRI 44, 501–510. 10.1053/j.sult.2023.10.006 37879545

